# A New Perspective on Cancer Therapy: Changing the Treaded Path?

**DOI:** 10.3390/ijms22189836

**Published:** 2021-09-11

**Authors:** Juliet N. E. Baidoo, Sumit Mukherjee, Khosrow Kashfi, Probal Banerjee

**Affiliations:** 1 Department of Chemistry, The College of Staten Island, City University of New York, Staten Island, NY 10314, USA; juliet.baidoo@csi.cuny.edu (J.N.E.B.); smukherjee@gradcenter.cuny.edu or; 2 Doctoral Program in Biochemistry, The Graduate Center of the City University of New York, New York, NY 10016, USA; 3 Department of Molecular, Cellular, and Biomedical Sciences, Sophie Davis School of Biomedical Education, City University of New York School of Medicine, New York, NY 10031, USA; kashfi@med.cuny.edu; 4 Graduate Program in Biology, City University of New York Graduate Center, New York, NY 10016, USA

**Keywords:** curcumin, GBM, iNOS, microglia, macrophages

## Abstract

During the last decade, we have persistently addressed the question, “how can the innate immune system be used as a therapeutic tool to eliminate cancer?” A cancerous tumor harbors innate immune cells such as macrophages, which are held in the tumor-promoting M2 state by tumor-cell-released cytokines. We have discovered that these tumor-associated macrophages (TAM) are repolarized into the nitric oxide (NO)-generating tumoricidal M1 state by the dietary agent curcumin (CC), which also causes recruitment of activated natural killer (NK) cells and cytotoxic T (Tc) cells into the tumor, thereby eliminating cancer cells as well as cancer stem cells. Indications are that this process may be NO-dependent. Intriguingly, the maximum blood concentration of CC in mice never exceeds nanomolar levels. Thus, our results submit that even low, transient levels of curcumin in vivo are enough to cause repolarization of the TAM and recruitment NK cells as well as Tc cells to eliminate the tumor. We have observed this phenomenon in two cancer models, glioblastoma and cervical cancer. Therefore, this approach may yield a general strategy to fight cancer. Our mechanistic studies have so far implicated induction of STAT-1 in this M2→M1 switch, but further studies are needed to understand the involvement of other factors such as the lipid metabolites resolvins in the CC-evoked anticancer pathways.

## 1. Introduction

Nitric oxide (NO) is produced from the amino acid arginine by the action of three types of nitric oxide synthases (NOS): neuronal NOS (nNOS or NOS1), inducible NOS (iNOS or NOS2), and endothelial NOS (eNOS or NOS3) [1]. nNOS occurs in specific brain neurons; iNOS can be induced by cytokines in a wide variety of cells, including innate immune cells such as microglia and macrophages to fight pathogens and cancer cells; and eNOS is expressed by the endothelial tissue, cardiac myocytes, platelets, certain brain neurons, syncytiotrophoblasts of the placenta, and kidney epithelial cells [2]. All three isoforms of NOS are dimeric enzymes that use L-arginine as the substrate and, with the help of molecular oxygen and nicotinamide-adenine dinucleotide phosphate (NADPH), convert this amino acid to L-citrulline and NO. Flavin adenine dinucleotide (FAD), flavin mononucleotide (FMN), and (6R-) 5,6,7,8-tetrahydro-L-biopterin (BH4) function as cofactors for all three NOS isozymes [3]. In this NOS complex, an electron transfer route is set up from NADPH through FAD and FMN to a heme unit held in the amino-terminal oxygenase domain, which also holds the cofactor BH4, molecular oxygen, and the main substrate L-arginine. At the heme center, the NADPH-derived electrons are used to set up the reduction of O_2_ and oxidation of L-arginine to L-citrulline and NO [4]. Intriguingly, non-immune cells such as endothelial cells, upon cytokine stimulation, have been shown to lyse tumor cells, possibly using NO [5]. This article focuses on iNOS, which is induced in stimulated macrophages that primarily kill microbes [6] but can also be used with appropriate adjustments to eliminate cancer cells. In a cancerous tumor, cytokines, such as IL10, secreted by the cancer cells, and the transcription factor STAT-3, cause induction of the competing enzyme arginase-1 (Arg1) in the tumor-associated microglia and macrophages (TAM). Arg1 catalyzes the degradation of L-arginine, thereby depleting iNOS of its substrate for NO production. Consequently, the TAM population is converted from the largely tumoricidal M1-type to the M2-type, suppressing the immune system and promoting tumor progression and metastasis [7,8]. In a series of studies conducted in syngeneic mouse models of glioblastoma (GBM) and cervical cancer, we have shown that the dietary agent curcumin (CC) causes repolarization of the M2-type TAM to the M1-type, thereby triggering a process that eliminates the tumor cells and tumor stem cells [9,10,11,12].

## 2. From the Anecdotal Use of a Remedy to the Clinic

The birth of quinine from the cinchona bark first gave us a treatment against malaria, although the underlying mechanisms of its action were revealed much later. Similarly, when Alexander Fleming discovered Penicillin from a mold, it gave an effective remedy against bacterial infections well before the mechanism of its action was delineated. However, over the years, the importance of biochemical mechanisms has gradually overtaken the significance of finding a therapeutic remedy. Thus, we think we know how metformin works in diabetes, but then we find that this same agent is also beneficial for many patients with Fragile X syndrome [13]. The possible mechanisms of its action in Fragile X syndrome are still being investigated. Consequently, if metformin had not already received FDA approval for diabetes, it would have had to undergo clinical trials as a new drug before being considered as a therapeutic for Fragile X syndrome. It is noteworthy that conducting a Phase I–III clinical trial is extremely expensive, further decreasing the chances of a new remedy reaching the patients who desperately need it.

For the past decade, the general approach in cancer therapy has been to apply genome-wide screening to determine the multiple mutations that develop in specific cells to trigger uncontrolled proliferation. Then, after surgical removal of a tumor, two therapeutic approaches are used: (1) administering antimetabolites (chemotherapeutic agents, CA) to eliminate fast-dividing (cancer?) cells, and (2) blocking mutated signaling proteins using synthetic drugs or monoclonal antibodies. Thus, the general approach involves a direct attack and killing of fast-dividing cancer (?) cells or mutant cells. Therefore, so far, any newly developed CA has been expected to acquire enough in vivo concentrations to kill cancer cells, and if it does not, then it is not expected to be a remedy.

Targeting signaling proteins that develop mutations has serious side effects because many normal cells also require those signaling proteins for their survival and function. Similarly, CAs most often cause immunosuppression by killing the normal immune cells or stimulating immunosuppressive myeloid cells [14,15,16]. Unfortunately, most tumors also harbor niches of rarely-dividing cancer stem cells [17,18], which are not killed by the CAs (antimetabolites) or radiation but are triggered to proliferate by radiation and signals from cell debris (discussed later) to cause tumor recurrence. Furthermore, these cancer stem cells express proteins that make them resistant to the CAs (termed as chemoresistance) [18,19]. Due to such difficulties, cancer therapy has not been able to keep pace with the revolutionized surgical techniques and detection strategies used currently. Consequently, many types of cancer, such as glioblastoma (GBM), pancreatic cancer, ovarian/endometrial cancer, and colon cancer, still have grim survival rates. Often the anticancer drugs are used with the desperate hope of gaining a few months of extra life to enjoy a seminal family event, perhaps.

## 3. Newly Appreciated Indirect Approaches in Cancer Therapy: Immunotherapy Using the Adaptive Immune System

During the past 20 years, it has become quite evident that our immune system could be used to overpower the varieties of cells that make a tumor thrive and proliferate. The first line of research focused on the adaptive immune system and successfully designed strategies to remove a cancer cell-mediated block on the cytotoxic T cells (Tc) with the hope that the Tc cells would then eliminate the cancer cells [20,21]. T-lymphocyte-associated antigen 4 (CTLA-4) and programmed cell death 1 (PD-1) are immune checkpoints that the Tc cells express to regulate them negatively. CTLA-4 is known to stop the potentially autoreactive T cells at the primary stage of naïve T-cell activation in the lymph nodes. By contrast, the PD-1 pathway is operational later, causing inhibition of already activated Tc cells. This pathway involves the Tc-expressed PD-1 (a receptor), which binds to the ligand PD-L1 expressed by the cancer cells [22].

**Figure 1:**
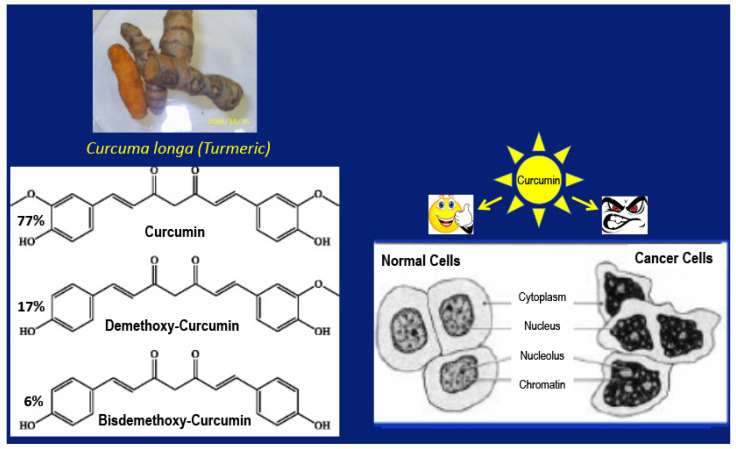
Curcumin occurs in the rhizome of the plant *curcuma longa* as a mixture of three closely related polyphenols and displays differential activity toward cancer cells and normal cells.

Inspired by the phenomenal success of inhibitors that block CTLA-4 and PD-1 in melanoma, pharmaceutical industries have been marketing three FDA-approved monoclonal antibody drugs, Yervoy (Ipilimumab, against CTLA-4), Keytruda (pembrolizumab, against PD-1), and Opdivo (nivolumab, against PD-1) [22,23,24]. However, immunotherapy has since then had little success in eliminating other cancers, including, for example, GBM, pancreatic and endometrial cancers. Thus, the oncologists are back to their long-practiced standard of care involving surgical removal of a tumor, followed by radiation and chemotherapy, even though this strategy is fraught with many invincible challenges. Amid such disappointing experiences, a new preclinical study has brought in renewed hope in immunotherapy. Lim and coworkers report that tumor necrosis factor-alpha (TNF-α) is a pivotal transducer of cancer cell-mediated suppression of Tc cell surveillance through the stabilization of PD-L1. Intriguingly, an inhibitor of ubiquitination and degradation of PD-L1, COP9 signalosome 5 (CSN5), is required for TNF-α-evoked stabilization of PD-L1. Unfortunately, CSN5 cannot be turned off entirely because its absence is lethal in mice [20]. However, the dietary agent CC (Figure 1) inhibits CSN5 without shutting it off to cause ubiquitination and degradation of PD-L1, thereby stimulating Tc cell surveillance [20].

The dietary agent CC has many interesting properties, and a salient feature among them is its ability to kill cancer cells but not normal cells (Figure 1). In the form isolated from the rhizomes of the plant *Curcuma longa*, CC comes as a mixture of curcumin (77%), dimethoxy-curcumin (17%), and *bis*-demethoxycurcumin (6%). Even though this polyphenol (CC) kills a large variety of cancer cells in culture [25], in the same concentration range, CC does not harm normal cells [26].

## 4. Immunotherapy Using the Innate Immune System

The second type of immunity is our “innate immunity,” readily available against a wide range of infectious agents and pathogens. It is generally not as massive as “adaptive immunity” but can be significantly stimulated by proper dietary components. This justifies the maxim circulating in the UK, “Eat your food as medicine or else you will have to consume medicine as food.” The recent COVID-19 pandemic has revealed intriguing infection rates and death patterns in different parts of the world. Scientists and physicians originally predicted that one of the largest slums in Asia, Dharavi in Mumbai, India, will become a hot spot for COVID-19 deaths. Although the positivity rate was 50% in Dharavi, disproving the “learned,” the death rate in this congested slum has been significantly lower than elsewhere, thus indicating that people living there have higher levels of immunity than those living under better sanitary conditions (https://theprint.in/theprint-otc/covid-prediction-model-shows-virus-is-washing-through-india-population-csirs-shekhar-mande/580035/, accessed on 8 September 2021). However, this may explain only the prophylactic nature of their immunity.

**Figure 2:**
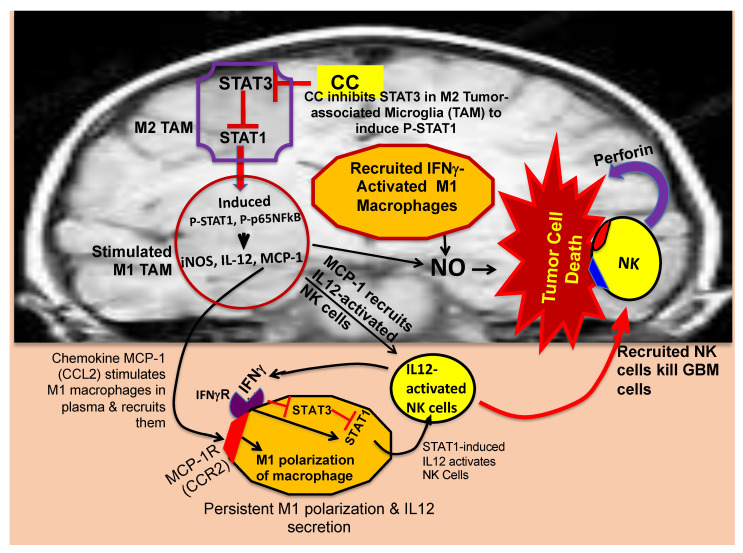
Proposed mechanism of M2→M1 repolarization of TAM, recruitment of NK cells, and elimination of GBM cells and GBM stem cells.

The current irking question is, “how can the innate immune system be used in a therapeutic approach to eliminate cancer?” During the last decade, research conducted by our group has revealed that a cancerous tumor harbors in its niches innate immune cells such as macrophages and microglia (in the brain), but predominantly in the tumor-promoting M2 state under the influence of tumor-released cytokines. Intriguingly, these tumor-associated macrophages (TAMs) are repolarized into the tumoricidal M1 state by the dietary agent curcumin (CC), which also causes recruitment of activated natural killer (NK) cells and Tc cells into the tumor, thereby launching an attack from inside to eliminate the tumor by killing both cancer cells as well as cancer stem cells (Figure 2) [9,11,12]. Furthermore, pharmacokinetic studies have revealed that oral delivery (gavage) of a lipid-complexed form of CC (curcumin phytosome) in rats yields plasma levels of 12 ng/mL (32 nM) of CC in 15 min, which drops off gradually while yielding curcumin glucuronide and curcumin sulfate, all of which disappear in 120 min, leaving behind minute quantities of tetrahydrocurcumin [27]. Similar studies performed by us have also revealed that the maximum blood concentration of CC in mice never exceeds nanomolar levels, which indicates that even low levels of curcumin are enough to cause repolarization and recruitment of M1 macrophages and activated NK cells as well as Tc cells to launch an attack on the tumor [9,10,11,12].

## 5. Proposed Mechanism of M2→M1 Repolarization of TAMs, Recruitment of Macrophages and NK Cells, and Tumor Elimination

Earlier reports show that the TAMs secrete the chemokine MCP-1 (also known as CCL2) [28] and that MCP-1 compromises the blood–brain barrier [29] to function as a chemoattractant for NK cells, macrophages, and T cells [8,30]. Based on such information, we suspected the involvement of MCP-1 in the recruitment of activated M1 macrophages and NK cells into the GBM tumor from the peripheral system, reviewed in [31]. We observed that phytosomal CC causes a dramatic induction of MCP-1 in the TAMs [12]. To explain the mechanistic steps, we showed that CC causes an inhibition of the transcription factor STAT-3 in the TAM (Figure 2). Since it is known that STAT-3 signaling via IL10 causes suppression of a second transcription factor STAT-1 [32], inhibition of STAT-3 elicits induction and activation of STAT-1. Our experimental findings confirmed CC-mediated suppression of STAT-3 and induction and activation of STAT-1 [12]. Activated STAT-1 causes induction of iNOS and the M1-specific cytokine IL12, which was also confirmed by our results. Meanwhile, the recruited M1 macrophages and existing M1 microglia in the GBM are both loaded with iNOS that catalyzes the release of NO, which would cause apoptosis of the GBM cells and GBM stem cells (Figure 2). Our experimental results also confirmed this possibility [12]. Additionally, activated NK cells are known to kill both M0 and M2-type macrophages and microglia, thereby amplifying the M1-type macrophages and microglia [33,34]. Therefore, the M1 polarization among the TAMs could be maintained by the recruited NK cells in our CC-treated GBM mouse brain [10]. The NK cells also form receptor-mediated immune synapses with GBM cells and GBM stem cells, thereby releasing perforin, which decimates the target cells (Figure 2) [34,35,36]. This was rigorously confirmed by our experiments using the GBM mouse model [12].

The TAMs can remain in multiple states of polarization. Still, a large number of research teams use a simpler model in which microglia and macrophages exist in “resting” M0, tumor-promoting and immunosuppressive M2, and anti-tumor or proinflammatory M1 states, with most literature evidence supporting the view that the M2-type constitutes the majority of the TAMs [37,38,39,40,41,42]. In fact, all these teams maintain that cytokines secreted by the tumor cells support the M2 state, and TAMs in the M2 state secrete factors that promote tumor progression and metastasis. Additionally, these articles have further established the importance of the strategy of M2→M1 repolarization of TAMs, which we had introduced in our earlier series of studies as a strategy to eliminate GBM and HPV(+)-cancer-cell-mediated tumors [9,10,11,12]. All tumors harbor tumor-associated immune cells such as TAM that can be repolarized to cause the elimination of both cancer cells as well as cancer stem cells. Thus, if developed correctly, this approach can yield a general strategy to fight many types of cancer.

Our group has used various forms of CC in these studies, including antibody-targeted CC, a commercially available phytosomal form of CC, and a synergistic formulation of CC along with other dietary polyphenols [9,10,11,12]. In addition, a small pharmaceutical company manufacturing curcumin-based pastilles was recently interested in conducting a Phase I/II clinical trial in GBM patients to investigate the efficacy of their product to cause repolarization of the TAMs. They have the support of a clinical group and documented experience of conducting other clinical trials with this clinical team. With appropriate funding, this collaborative effort could achieve feasibility.

## 6. Investigating Ways to Use the Innate Immune System in Cancer Therapy Requires the Use of Immune-Competent Animal Models

It is understood that the innate immune system weakens with age. The possible role of the innate immune system in preventing tumor formation and cancer progression is thus supported by the experience that cancer incidence increases with age. Unfortunately, this important clue has been largely overlooked and, therefore, overshadowed by the widespread belief that any CA can be successful if it can directly kill the cancer cells in a subject. Consequently, most preclinical studies have used human cancer cells in their in vivo studies but only in immune-compromised mice to study the effect of the CA to eliminate the grafted human cancer cells. Thus, the influence of the immune system in cancer cell elimination has been largely overlooked.

**Figure 3:**
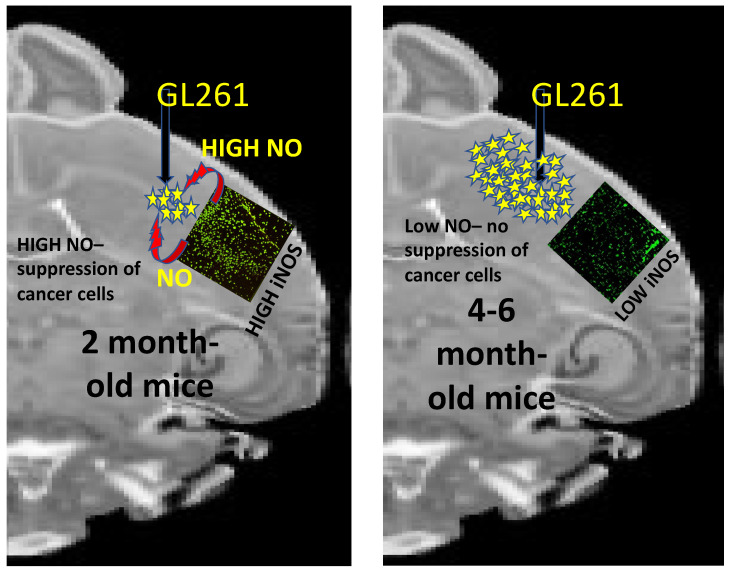
The likely reason for tumor resistance in the brain of 2-month-old mice (The stars represent GL261 GBM cells).

To study the whole organism along with its arsenal of anticancer armaments, we have always used immune-competent C57BL/6 mice and mouse GBM or melanoma cells to construct a syngeneic mouse model. During the development of the GBM mouse model, we learned a few lessons that highlighted the importance of age and the innate immune system in establishing cancer in an organism. While standardizing conditions to generate the syngeneic C57BL/6 mouse model of GBM, we observed that to consistently form a GBM brain tumor after orthotopic implantation of 10^5^ GL261 (mouse GBM) cells, we had to use mice that were 4 months or older. In this GBM mouse model, we also observed the formation of a tumor that occupied 5–10% of brain volume on day 11 after implantation. Subsequently, when left untreated, the tumor grew rapidly to occupy most of the brain by day 25, causing morbidity and death of the mouse. Quite strikingly, implantation of 10^5^ GL261 cells in the same mice at 2 months of age did not produce any tumor. In our past studies, while looking into the polarization of tumor-associated microglia, we were not able to go deeper into the possible reasons why each 2-month-old mouse did not form a tumor with 10^5^ GL261 cells implanted in its brain [9,10,12,20]. Our recent unpublished studies have now shown that the brain’s implantation area displays high levels of iNOS-expressing Iba1+ microglia in drug-naïve, 2-month-old mice (Figure 3). This was in sharp contrast to the low expression of iNOS observed in the tumor-associated microglia in the vehicle-treated, 4–6-month-old mice in our earlier studies [9,10,12,20]. Such observation suggests a major involvement of the innate immune cells in keeping most younger individuals (or organisms) cancer-free (Figure 3). Our future studies will systematically study the age-dependence of polarization of the innate immune system to understand why aging people become more prone to cancer.

## 7. Nitric Oxide (NO) Production by Innate Immune Cells

An important aspect of such processes appears to be the levels and tissue distribution of the gaseous signaling molecule NO. This highly transient molecule with an extravascular half-life of 90 milliseconds to 2 s [43] is believed to play important but varying roles in cellular functions at different concentrations (reviewed in [44]). Thus, at low concentrations (100 nM), it is believed to cause proliferation and angiogenesis; at intermediate concentrations (100–500 nM), it is understood to promote invasiveness, metastasis, cytoprotection, suppression of apoptosis, and cancer promotion; at high concentrations (>500 nM), NO is reported to cause DNA damage, cytotoxicity, and apoptosis. Being a highly transient molecule, how NO achieves such local concentrations may need further studies. Since it is known that NO is produced by both innate immune cells and cancer cells, a critical question arises: “what type or types of cells yield this NO concentration”? In our studies in the GBM and HPV mouse models, we realize that our experiments were limited to analyzing the relative expression levels of inducible nitric oxide synthase (iNOS) in the microglia and macrophages [9,10,11,12]. We investigated how this process marked the repolarization of the iNOS^Low^/ Arginase-1^High^ M2-type microglia/macrophages to the iNOS^Hgh^/ Arginase-1^Low^ M1-type innate immune cells. However, the NO signaling mechanism would be better-defined if we could include appropriate strategies to measure the NO produced by these TAM and not the NO produced by the cancer cells and the endothelial cells; while in vitro studies could achieve this task, we would still be in the dark about the rate of NO synthesis by the microglia/macrophages in the presence of cell–cell interactions with the surrounding cells in the in vivo system. Our future studies will address this question through exploratory experiments.

## 8. Possible Mechanisms of Synergism among Curcumin and Other Polyphenols

**Figure 4:**
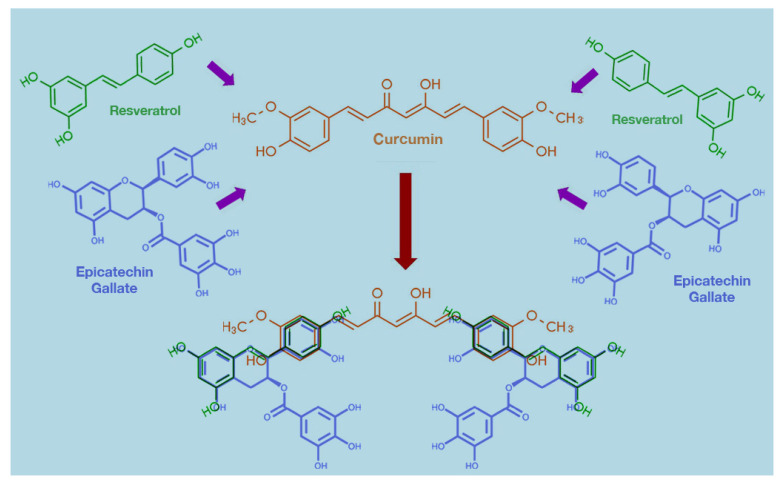
Proposed pi-complex formation among the polyphenols curcumin, resveratrol, and epicatechin gallate in Tricurin due to hydrophobic association in water.

In our initial studies, we identified CC as the agent that would bring about this M2→M1 repolarization of the TAMs [9,12], but soon after that, we discovered that other dietary polyphenols such as resveratrol (Res) and epicatechin gallate (ECG) could form a synergistic combination with CC that was more potent than CC in eliminating a variety of cancer cells both in vitro and in vivo [45]. It was realized that this specific combination in the molar ratio CC:ECG:Res:4:1:12.5 was able to stabilize CC and make the formulation more water-soluble because of the water solubility of ECG. We hypothesized that this occurred through pi-electron stacking among the hydrophobic aromatic rings in the three compounds (Figure 4). However, we realized later that such synergistic potentiation of anticancer activity was not only due to stabilization of CC but also due to a significantly higher potency of Tricurin than CC in causing the M2→M1 repolarization of the TAMs and recruitment of NK cells into the tumor in the mouse models of GBM and cervical cancer [10,11].

## 9. Chemoresistance Resulting from CA Treatment of Cancer Cells

Although the current standard of care for GBM involves high order of precision in debulking the GBM, an almost invincible challenge is that some GBM cells of some type evade the surgical procedure. To meet this challenge, the standard of care also includes subsequent chemotherapy and radiation therapy. As discussed earlier, unfortunately, despite all efforts, tumor recurrence is a common phenomenon due to the presence of GBM stem-like cells that evade such initial therapy [46]. Our discussions in the following sections mention the possible involvement of the GBM stem cells in this challenging problem. Still, many studies have pointed to some specialized GBM stem-like cells instead of or in addition to the GBM stem cells [46]. Such stem-like cells, named as “recurrence-initiating stem-like cancer (RISC) cells”, are more aggressive than the GBM stem cells and can trigger a recurrence of GBM, which is consistent with the shorter survival of patients [47]. Furthermore, unlike the GBM stem cells, these cells lack the stem cell marker CD133 but display high levels of CD15, BMI1, and SOX2 [48]. Therefore, it would be imperative to study if the TAM and the recruited NK cells can eliminate the RISC cells.

In cancer therapy, serious side effects have been noted for all CAs at higher doses. For example, doxorubicin’s most dangerous side effect is dilated cardiomyopathy, leading to congestive heart failure. It is observed with 4% incidence at a dose of 500–550 mg/m^2^, with 18% incidence at 551–600 mg/m^2^, and with 36% incidence at ≥600 mg/m^2^ [49]. Mainly, three interlinked phenomena contribute to the side effects of CAs: (a) the cancer cells develop resistance to the CAs during treatment [17,50]; (b) such chemoresistance forces the use of higher doses of a CA; (c) the higher doses of the CA also kill the proliferating immune cells, thus causing immuno-suppression [14,15]. Thus, a safe strategy that can eliminate the cancer cells’ chemoresistance and stimulate tumoricidal immune cells (e.g., microglia/macrophages and NK cells) could dramatically improve the outcome of cancer therapy [9,12]. Curcumin (CC) eliminates chemo- and radioresistance [51,52,53,54], rendering cancer cells more sensitive to CAs. Additionally, we have shown that appropriately delivered CC primes the innate immune system, killing cancer cells and cancer stem cells [9,10,11,12,55].

Prior studies have demonstrated that multiple proteins can be responsible for chemoresistance in various cancer cell types. A few of such chemoresistance-causing proteins that are induced during CA treatment include: (a) the ubiquitin ligase MDM2 that ubiquitinates the cell cycle-blocking transcription factor p53 [53]; (b) the thymidine phosphorylase, which is base Excision Repair Cross-complementary protein ERCC1 [56]; (c) O-6-methylguanine-DNA methyltransferase (MGMT) [57]; and (d) the multidrug resistance (MDR) proteins [58]. The studies cited here show that CC suppresses the expression of MDM2, ERCC1, and MGMT but inhibits the activity of the MDR proteins by binding to the drug-binding sites of these ATP-dependent transporters that expel chemotherapeutic drugs from cell membranes and the blood–brain barrier (BBB) [54]. Although most of these proteins have been linked to chemoresistance, it is rare that the overall chemoresistance of a cancer cell type is built by significant contributions from several proteins. Instead, a major contribution to chemoresistance is most commonly observed from the family of ATP-dependent drug-transporters termed as ABC transporters, which are MDR proteins [59,60]. For example, CA treatment has been demonstrated to cause induction of ABCB1, which belongs to this family of ATP-dependent drug transporters [61]. Intriguingly, earlier publications show that CC inhibits the MDR proteins at nanomolar concentrations in cell culture [54,58], whereas inhibition of the other chemoresistance-causing proteins requires micromolar concentrations of CC [53,56,57]. In view of the CC-mediated influence on the TAMs and reports showing dramatic chemo-sensitizing effects of CC, relevant in vivo studies are required to explore the impact of the TAMs and NO on the chemoresistance-causing proteins.

## 10. The Role of Curcumin in Context-Dependent Regulation of iNOS and the Involvement of Resolvins in Inflammation and Tumor Clearance

As observed by our group and others, in the tumor microenvironment, CC evokes a dramatic change by causing M2→M1 transition and induction in iNOS in the tumor-associated microglia/macrophages (TAM) [9,12]. However, intriguingly, in the absence of a tumor, CC causes an inhibition of the proinflammatory M1-type macrophages and iNOS [62,63,64,65]. The mechanistic details of this dual role of CC are currently being investigated, but the involvement of the lipid metabolites, resolvins, may provide a clue. Commonly known mediators of inflammation are tumor necrosis factor (TNF) and interleukins [66] and the lipid mediators eicosanoids and thromboxanes [67,68], which signal during the onset phase of inflammation after a trauma or tissue injury [69], thereby increasing vascular permeability, promoting infiltration of neutrophils, and removing dead cells. Other lipid mediators prevent uncontrolled inflammation during the resolution phase of inflammation [70,71] and repolarize the proinflammatory M1-type macrophages at the injury site to the anti-inflammatory M2 phenotype [72]. Could CC be involved in this M1→M2 transition during the resolution phase?

The family of lipid mediators eicosapentaenoic acid (EPA) and docosahexaenoic acid (DHA) are believed to function through their metabolites “resolvins” as anti-inflammatory signals [73,74]. EPA and DHA are n-3 polyunsaturated fatty acids (n-3 PUFA) that are produced at the peak of inflammation. These molecules are metabolized to resolvins, which signal through specific G protein-coupled receptors to trigger the resolution phase of inflammation [69]. It has been shown that peroxidation of n-3 PUFA is associated with a block of iNOS induction at the gene expression level under proinflammatory conditions [75]. Intriguingly, CC has been shown to boost the synthesis of DHA from α-linolenic acid (ALA) by elevating the levels of enzymes involved in the steps of elongation (elongase 2) of ALA and desaturation (FADS2) to produce DHA [76]. Thus, CC functions through DHA to block iNOS induction during inflammation. It also leads to the formation of the DHA metabolites resolvins, which have been reported to block tumor recurrence [77]. These studies also reveal that the lipids such as phosphatidylserine, which are exposed on the surface of CA-generated apoptotic cells in the debris, trigger tumor regrowth from the residual, live cancer cells. The resolvins function through their receptors to prevent tumor cell debris-evoked cancer recurrence, and this has been considered an important topic in cancer biology [77]. Since most CAs generate tumor cell debris, the resolvins or their downstream signaling partners could be crucial in future tumor therapy. Quite strikingly, resolvin-evoked signaling also influences the innate immune cells and CC-mediated regulation of signaling proteins, cytokines, and NK cells [77,78,79]. Thus, resolvins also increase the anticancer activity of CC and protect activated NK cells that eliminate cancer cells. As for the in vivo effects of such innate immune cells in our syngeneic GBM mouse model, CC was observed to cause a suppression of the M2-linked transcription factor STAT-3, induction of the proinflammatory cytokine IL12, and activation of the M1-linked transcription factor STAT-1 in the TAMs and activation of NK cells to cause elimination of GBM (Figure 2) [9,12]. This was also consistent with earlier reports showing that alternatively activated M2-like TAMs, which are known to harbor induced IL10 rather than induced IL12, promote tumor progression, whereas the IL12-expressing M1-type TAMs are tumoricidal [7,80]. The studies on the resolvins have also revealed that the resolvins stimulate signaling pathways that eventually cause suppression of this tumor reappearance and stimulation of macrophages to carry out efferocytosis of apoptotic cells in the debris [77]. However, these studies were conducted in the context of peripheral tumors. Since GBM is isolated from the immune cells in the peripheral system, the implications of such resolvin-signaling mechanisms in the context of GBM may require more careful analyses. Clearance of tumor debris is a difficult problem, but in our GBM model, we have observed only a small presence of scar tissue but never had any encounter with accumulated cell debris in mice fully rescued from GBM. This prompts us to believe that a mechanism of clearance of cell debris is in operation in our mouse model of GBM. Based on a study in the peripheral system using a human papilloma virus-based animal model, we know that, like CC, resolvin D1 also causes an induction of monocytic MCP-1 and recruitment of classically activated (M1-type) anti-tumor monocytes into the tumor [81]. Thus, in view of our studies in mouse models of HPV and GBM [9,11,12], resolvins may play a synergistic role with CC in the clearance of both GBM and HPV-linked tumors. Our future studies will explore the possible involvement of resolvins in the CC-mediated rescue of the syngeneic GBM mice.

## 11. Patents

“Novel Curcumin-antibody Conjugates as Anti-Cancer Agents”. Banerjee, P. (PI) and Raja, K. 1038-58 PCT/US/Approved 2016.

## Data Availability

Data included in this review were obtained from articles listed on the NIH National Library of Medicine site: https://www.ncbi.nlm.nih.gov/myncbi/probal.banerjee.1/bibliography/public/ (accessed on 8 September 2021).
